# The chromosomal genome sequence of a shovel-head worm, *Maea johnstoni* (Fiege, Licher & Mackie, 2000) (Spionida: Magelonidae) and its associated microbial metagenome sequences

**DOI:** 10.12688/wellcomeopenres.27132.1

**Published:** 2026-07-21

**Authors:** Kate Mortimer, José M. Martín-Durán, Graeme Oatley, Elizabeth Sinclair, Eerik Aunin, Noah Gettle, Camilla Santos, Michael Paulini, Haoyu Niu, Victoria McKenna, Rebecca O’Brien, Wellcome Sanger Institute Tree of Life Management, Samples and Laboratory team, Wellcome Sanger Institute Scientific Operations: Sequencing Operations, Wellcome Sanger Institute Tree of Life Core Informatics team, EBI Aquatic Symbiosis Genomics Data Portal Team, Aquatic Symbiosis Genomics Project Leadership

**Affiliations:** 1 Natural Sciences Amgueddfa Cymru – Museum Wales, Cardiff, Wales, UK; 2 School of Biological and Behavioural Sciences, Queen Mary University of London, London, England, UK; 3 Tree of Life Programme, Wellcome Sanger Institute, Hinxton, England, UK

**Keywords:** Maea johnstoni, shovel-head worm, genome sequence, chromosomal, Spionida, microbial metagenome assembly

## Abstract

We present a genome assembly from a specimen of *Maea johnstoni* ( Fiege, Licher & Mackie, 2000) (shovel-head worm; Annelida; Polychaeta; Spionida; Magelonidae). The genome sequence has a total length of 881.61 megabases. Most of the assembly (97.37%) is scaffolded into 18 chromosomal pseudomolecules. The mitochondrial genome has also been assembled, with a length of 15.47 kilobases. From the metagenome data, we recovered two Spirochaetota bins, one of which was a MAG.

## Species taxonomy

Eukaryota; Opisthokonta; Metazoa; Eumetazoa; Bilateria; Protostomia; Spiralia; Lophotrochozoa; Annelida; Polychaeta; Sedentaria; Canalipalpata; Spionida; Magelonidae; *Maea*; *Maea johnstoni* ( Fiege, Licher & Mackie, 2000) (NCBI:txid3698974).

## Background

The shovel-head worm *Maea johnstoni*, a magelonid polychaete, occurs around the UK, the North Sea, and the English Channel down to the Mediterranean ( Fiege *et al.*, 2000). It occurs intertidally and subtidally at depths of 0–100 m. It can reach high densities onshore, e.g., Berwick-upon Tweed, north-east England. The species is known from sandy sediments ( Fiege *et al.*, 2000; Mackie *et al.*, 1995, 2006; Meißner & Darr, 2009; Robinson *et al.*, 2009) and occurs in sympatry with other shovel-head worm species, often *Maea mirabilis* Johnston, 1865, with which it shares many morphological features.

Shovel-head worms are slender worms, easily recognised by a shovel-shaped head region, two long papillated palps inserted on the ventral surface on either side of the buccal region, and a body divided into two regions: a thorax of eight or nine chaetigers bearing capillary chaetae, and an abdomen of many chaetigers carrying hooded hooks that terminates with the pygidium ( Mortimer, 2019; Mortimer *et al.*, 2021b; Parapar *et al.*, 2021). Their slender nature means they are often incomplete when collected, and the delicate palps may be missing.

*Maea johnstoni* can be distinguished from other shovel-head worm species using the following characters: the presence of an elongate and rounded prostomium; a thorax of nine chaetigers; foliaceous thoracic notopodial lamellae, the upper surfaces of which carry elk-horn-shaped crenulations; superior dorsal lobes on chaetigers 4 to 8; both anteriorly and posteriorly open abdominal lateral pouches, and tridentate abdominal hooded hooks oriented unidirectionally within each ramus ( Fiege *et al.*, 2000; Mortimer *et al.*, 2020, 2021a; Mortimer & Mackie, 2014; Taylor & Mortimer, 2023).

The abdominal lateral pouches of *M. johnstoni* are a helpful identification feature ( Mortimer & Mackie, 2014). Those of the anterior abdomen are anteriorly open and consist of a dorsal and ventral flap with a convoluted membrane in between. They are paired, occurring on either side of a chaetiger. Those towards the mid and posterior abdomen are posteriorly open, appearing as simple C-shaped pocket-like folds. They occur singularly, alternating from one side of the body to the other. The function of these pouches is currently unknown ( Mortimer & Mackie, 2014).

Very little is known about the reproduction of shovel-head worms. However, the eggs and sperm masses are easily observed within the abdominal cavity when present, allowing the separation of male and female worms. Fiege *et al.* (2000) noted that eggs are approximately 125 μm in size. Published works suggest spawning may occur between May and August ( Mortimer & Mackie, 2014) in European waters. Wilson (1982) described magelonid larval development for the morphologically similar *M. mirabilis*, but no further information is available for *M. johnstoni.* Whilst Mortimer & Mackie (2014) observed intermittent lateral sinuous movements of the thorax of *M. johnstoni* outside of the burrow, postulating a link with reproductive activity, no further conclusions were made. The morphologically similar species *Maea sacculata* (Hartman, 1961) from north-west America is considered an annual species ( Fauchald, 1983), and Mortimer (2019) noted that laboratory-kept *M. johnstoni* animals lived for approximately 2 years.

*Maea johnstoni* is an active species that burrows more or less continuously through the sediment using an eversible burrowing organ ( Mortimer & Mackie, 2014). It is primarily considered a surface deposit feeder that uses its papillated palps to collect food particles from the surface of the sediment, which are transferred to the mouth by cooperative movements of the palp papillae ( Mortimer & Mackie, 2014; Parapar *et al.*, 2021).

*Maea johnstoni* is a common species, and is not considered to be under threat. COI, 16S and 28S sequences for the species were included in a project investigating species off western Africa ( Mortimer *et al.*, 2022, BOLD Process ID ICBA376–17).

## Methods

### Sample acquisition

The specimen used for genome sequencing was an adult *Maea johnstoni* (specimen ID QMOUL0000200, ToLID wdMagJohn1; Figure 1), collected from Cawsands Bay, Plymouth, United Kingdom (latitude 50.3296, longitude 4.1998) on 2022-06-14. The specimen was collected and identified by Kate Mortimer (Department of Natural Sciences, Amgueddfa Cymru, Cardiff, United Kingdom). A second *M. johnstoni* specimen was used for Hi-C sequencing (specimen ID QMOUL0000201, ToLID wdMagJohn2) and a third (specimen ID MBA-220614-002D, ToLID wdMagJohn15) for RNA sequencing. These specimens were collected from Cawsands Bay, Plymouth, UK (latitude 50.3296, longitude 4.1998) on 2022-06-14.

**Figure 1.**
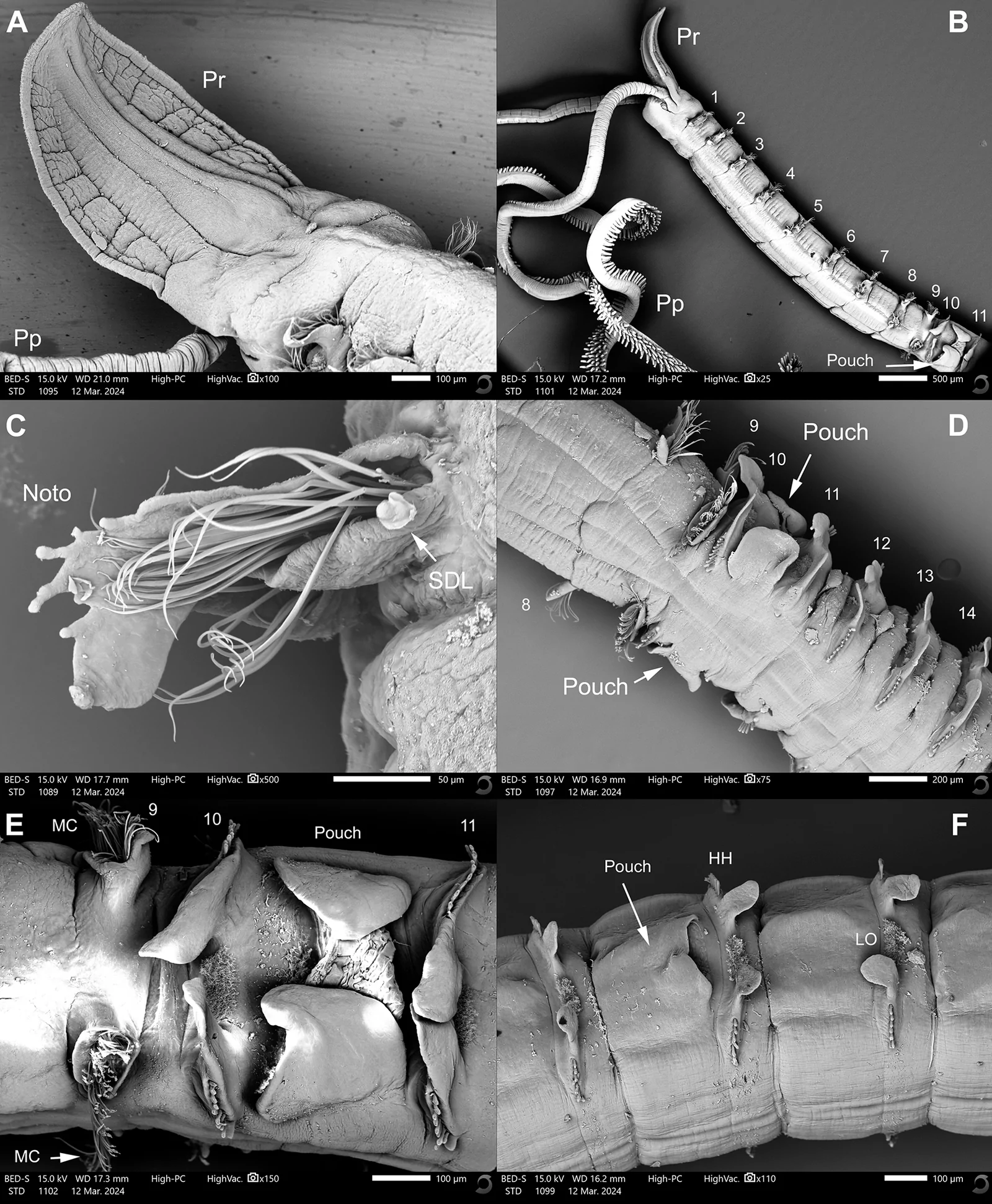
Scanning Electron Microscopy images of *Maea johnstoni* – A) Shovel-shaped prostomium (dorsal view); B) Thorax (lateral view); C) Thoracic notopodial lamellae with elk-horn crenulations; D) Thorax/Abdomen junction (ventral view) showing paired anteriorly open abdominal lateral pouches; E) chaetigers 9–11 (lateral view) showing anteriorly open pouch between chaetigers 10–11 and specialised (mucronate) chaetae of chaetiger 9; F) abdominal chaetigers (lateral view) showing unpaired posteriorly open pouch. HH = hooded hook, LO = lateral organs, MC = mucronate chaetae, Noto – notopodium, Pp = palp, Pr = prostomium, SDL = Superior Dorsal Lobe. Numbers indicate chaetiger number.

### Nucleic acid extraction

Protocols for high molecular weight (HMW) DNA extraction developed at the Wellcome Sanger Institute (WSI) Tree of Life Core Laboratory are available on protocols.io ( Howard *et al.*, 2025). The wdMagJohn1 sample was weighed and triaged to determine the appropriate extraction protocol. Tissue from the whole organism was homogenised by powermashing using a PowerMasher II tissue disruptor. HMW DNA was extracted using the Manual MagAttract protocol. DNA was sheared into an average fragment size of 12–20 kb following the Megaruptor®3 for LI PacBio protocol. Sheared DNA was purified by automated SPRI (solid-phase reversible immobilisation). The concentration of the sheared and purified DNA was assessed using a Nanodrop spectrophotometer and Qubit Fluorometer using the Qubit dsDNA High Sensitivity Assay kit. Fragment size distribution was evaluated by running the sample on the FemtoPulse system. For this sample, the final post-shearing DNA had a Qubit concentration of 11.2 ng/μL, with a fragment size of 13.4 kb. The 260/280 spectrophotometric ratio was 1.75, and the 260/230 ratio was 2.07.

RNA was extracted from mid-body tissue of the wdMagJohn15 specimen in the Tree of Life Laboratory at the WSI using the RNA Extraction: Automated MagMax™ *mir*Vana protocol. The RNA concentration was assessed using a Nanodrop spectrophotometer and a Qubit Fluorometer using the Qubit RNA Broad-Range Assay kit. Analysis of the integrity of the RNA was done using the Agilent RNA 6000 Pico Kit and Eukaryotic Total RNA assay.

### PacBio HiFi library preparation and sequencing

Library preparation and sequencing were performed at the WSI Scientific Operations core. Libraries were prepared using the SMRTbell Prep Kit 3.0 (Pacific Biosciences, California, USA), following the manufacturer’s instructions. The kit includes reagents for end repair/A-tailing, adapter ligation, post-ligation SMRTbell bead clean-up, and nuclease treatment. Size selection and clean-up were performed using diluted AMPure PB beads (Pacific Biosciences). DNA concentration was quantified using a Qubit Fluorometer v4.0 (ThermoFisher Scientific) and the Qubit 1X dsDNA HS assay kit. Final library fragment size was assessed with the Agilent Femto Pulse Automated Pulsed Field CE Instrument (Agilent Technologies) using the gDNA 55 kb BAC analysis kit.

The sample was sequenced using the Sequel IIe system (Pacific Biosciences, California, USA). The concentration of the library loaded onto the Sequel IIe was in the range 40–135 pM. SMRT Link software, a PacBio web-based workflow manager, was used to set up and monitor the run, and to perform primary and secondary analysis of the data upon completion.

### Hi-C

***Sample preparation and crosslinking***

The Hi-C sample was prepared from 20–50 mg of frozen whole organism tissue from the wdMagJohn20 sample using the Arima-HiC v2 kit (Arima Genomics). Following the manufacturer’s instructions, tissue was fixed and DNA crosslinked using TC buffer to a final formaldehyde concentration of 2%. The tissue was homogenised using the Diagnocine Power Masher-II. Crosslinked DNA was digested with a restriction enzyme master mix, biotinylated, and ligated. Clean-up was performed with SPRISelect beads before library preparation. DNA concentration was measured with the Qubit Fluorometer (Thermo Fisher Scientific) and Qubit HS Assay Kit. The biotinylation percentage was estimated using the Arima-HiC v2 QC beads.

***Hi-C library preparation and sequencing***

Biotinylated DNA constructs were fragmented using a Covaris E220 sonicator and size selected to 400–600 bp using SPRISelect beads. DNA was enriched with Arima-HiC v2 kit Enrichment beads. End repair, A-tailing, and adapter ligation were carried out with the NEBNext Ultra II DNA Library Prep Kit (New England Biolabs), following a modified protocol where library preparation occurs while DNA remains bound to the Enrichment beads. Library amplification was performed using KAPA HiFi HotStart mix and a custom Unique Dual Index (UDI) barcode set (Integrated DNA Technologies). Depending on sample concentration and biotinylation percentage determined at the crosslinking stage, libraries were amplified with 10–16 PCR cycles. Post-PCR clean-up was performed with SPRISelect beads. Libraries were quantified using the AccuClear Ultra High Sensitivity dsDNA Standards Assay Kit (Biotium) and a FLUOstar Omega plate reader (BMG Labtech).

Prior to sequencing, libraries were normalised to 10 ng/μL. Normalised libraries were quantified again to create equimolar and/or weighted 2.8 nM pools. Pool concentrations were checked using the Agilent 4200 TapeStation (Agilent) with High Sensitivity D500 reagents before sequencing. Sequencing was performed using paired-end 150 bp reads on the Illumina NovaSeq 6000.

### RNA library preparation and sequencing

Libraries were prepared using the NEBNext® Ultra II Directional RNA Library Prep Kit for Illumina (New England Biolabs), following the manufacturer’s instructions. Poly(A) mRNA was isolated from 100 ng of total RNA using oligo (dT) beads, converted to cDNA, and uniquely indexed; 14 PCR cycles were performed. Libraries were prepared with an intended fragment size of 100–300 bp. Libraries were quantified, normalised, and pooled equimolarly to a final concentration of 2.8 nM before dilution to 150 pM for loading. Sequencing was carried out on the Illumina NovaSeq X, generating paired-end reads.

### Genome assembly

Prior to assembly of the PacBio HiFi reads, a database of *k*-mer counts ( *k* = 31) was generated from the filtered reads using FastK. GenomeScope2 ( Ranallo-Benavidez *et al.*, 2020) was used to analyse the *k*-mer frequency distributions, providing estimates of genome size, heterozygosity, and repeat content.

The HiFi reads were assembled using Hifiasm ( Cheng *et al.*, 2021) with the --primary option. Haplotypic duplications were identified and removed using purge_dups ( Guan *et al.*, 2020). The Hi-C reads ( Rao *et al.*, 2014) were mapped to the primary contigs using bwa-mem2 ( Vasimuddin *et al.*, 2019), and the contigs were scaffolded in YaHS ( Zhou *et al.*, 2023) with the --break option for handling potential misassemblies. The scaffolded assemblies were evaluated using Gfastats ( Formenti *et al.*, 2022), BUSCO ( Manni *et al.*, 2021) and MERQURY.FK ( Rhie *et al.*, 2020).

The mitochondrial genome was assembled using MitoHiFi ( Uliano-Silva *et al.*, 2023). The MitoHiFi reference was the mitochondrial genome of *Magelona mirabilis* (NC_028711.1).

### Assembly curation

The assembly was decontaminated using the Assembly Screen for Cobionts and Contaminants ( ASCC) pipeline. TreeVal was used to generate the flat files and maps for use in curation. Manual curation was conducted primarily in PretextView and HiGlass ( Kerpedjiev *et al.*, 2018). Scaffolds were visually inspected and corrected as described by Howe *et al*. (2021). Manual corrections included 325 breaks, 531 joins, and removal of 68 haplotypic duplications. This reduced the scaffold count by 60.2%, increased the scaffold N50 by 5.9%, and reduced the total assembly length by 7.3%. The curation process is described at sanger-tol/curation-resources . PretextSnapshot was used to generate a Hi-C contact map of the final assembly.

### Assembly quality assessment

The Merqury.FK tool ( Rhie *et al.*, 2020) was run in a Singularity container ( Kurtzer *et al.*, 2017) to evaluate *k*-mer completeness and assembly quality for the primary and alternate haplotypes using the *k*-mer database ( *k* = 31) computed prior to genome assembly. The analysis outputs included assembly QV scores and completeness statistics.

The genome was analysed using the BlobToolKit pipeline, a Nextflow implementation of the earlier Snakemake version ( Challis *et al.*, 2020). The pipeline aligns PacBio reads using minimap2 ( Li, 2018) and SAMtools ( Danecek *et al.*, 2021) to generate coverage tracks. It runs BUSCO ( Manni *et al.*, 2021) using lineages identified from the NCBI Taxonomy ( Schoch *et al.*, 2020). For the three domain-level lineages, BUSCO genes are aligned to the UniProt Reference Proteomes database ( Bateman *et al.*, 2023) using DIAMOND blastp ( Buchfink *et al.*, 2021). The genome is divided into chunks based on the density of BUSCO genes from the closest taxonomic lineage, and each chunk is aligned to the UniProt Reference Proteomes database with DIAMOND blastx. Sequences without hits are chunked using seqtk and aligned to the NT database with blastn ( Altschul *et al.*, 1990). The BlobToolKit suite consolidates all outputs into a blobdir for visualisation. The BlobToolKit pipeline was developed using nf-core tooling ( Ewels *et al.*, 2020) and MultiQC ( Ewels *et al.*, 2016), with containerisation through Docker ( Merkel, 2014) and Singularity ( Kurtzer *et al.*, 2017).

### Metagenome assembly

The metagenome assembly was generated using MetaMDBG ( Benoit *et al.*, 2024) and binned usingMetaBAT2 ( Kang *et al.*, 2019). The resulting bin sets of each binning algorithm were optimised and refined using DAS Tool ( Sieber *et al.*, 2018). PROKKA ( Seemann, 2014) was used to identify tRNAs and rRNAs in each bin, CheckM ( Parks *et al.*, 2015) (checkM_DB release 2015-01-16) was used to assess bin completeness/contamination, and GTDB-Tk ( Chaumeil *et al.*, 2022) (GTDB release 214) was used to taxonomically classify bins. Taxonomic replicate bins were identified using dRep ( Olm *et al.*, 2017) with default settings (95% ANI threshold). All bins were assessed for quality and categorised as metagenome-assembled genomes (MAGs) if they met the following criteria: contamination ≤5%, presence of 5S, 16S, and 23S rRNA genes, at least 18 unique tRNAs, and either ≥90% completeness or ≥50% completeness with fully circularised chromosomes ( Bowers *et al.*, 2017). Bins that did not meet these thresholds, or were identified as taxonomic replicates of MAGs, were retained as ‘binned metagenomes’ provided they had ≥50% completeness and ≤10% contamination.

## Genome sequence report

### Sequence data

PacBio sequencing of the *Maea johnstoni* specimen generated 20.91 Gb (gigabases) from 2.32 million reads, which were used to assemble the genome. GenomeScope2.0 analysis estimated the haploid genome size at 509.68 Mb, with a heterozygosity of 4.43% and repeat content of 45.75%. These estimates guided expectations for the assembly. Based on the estimated genome size, the sequencing data provided approximately 34× coverage. Hi-C sequencing produced 138.63 Gb from 459.05 million reads, which were used to scaffold the assembly. RNA sequencing data were also generated and are available in public sequence repositories. Table 1 summarises the specimen and sequencing details.

**Table 1.** Specimen and sequencing data for *Maea johnstoni* (BioProject PRJEB71422).

Platform	PacBio HiFi	Hi-C	RNA-seq
**ToLID**	wdMagJohn1	wdMagJohn20	wdMagJohn15
**Specimen ID**	QMOUL0000200	MBA-220614-002I	MBA-220614-002D
**BioSample (source individual)**	SAMEA111454427	SAMEA112148702	SAMEA111562193
**BioSample (tissue)**	SAMEA111454439	SAMEA112152788	SAMEA111562417
**Tissue**	whole organism	whole organism	mid-body
**Instrument**	Sequel IIe	Illumina NovaSeq 6000	Illumina NovaSeq X
**Run accessions**	ERR12408773	ERR12512722	ERR13093652
**Read count total**	2.32 million reads	459.05 million read pairs	89.75 million read pairs
**Base count total**	20.91 Gb	138.63 Gb	27.11 Gb

### Assembly statistics

The primary haplotype was assembled, and contigs corresponding to an alternate haplotype were also deposited in INSDC databases. The final assembly has a total length of 881.61 Mb in 1 250 scaffolds, with 8 735 gaps, and a scaffold N50 of 46.58 Mb ( Table 2).

**Table 2.** Genome assembly data for *Maea johnstoni.*

Genome assembly	Primary assembly
**Assembly name**	wdMagJohn1.1
**Assembly accession**	GCA_963942565.1
**Alternate haplotype accession**	GCA_963942585.1
**Assembly level**	chromosome
**Span (Mb)**	881.61
**Number of chromosomes**	18
**Number of contigs**	9 985
**Contig N50**	0.14 Mb
**Number of scaffolds**	1 250
**Scaffold N50**	46.58 Mb
**Organelles**	Mitochondrial genome: 15.47 kb

The scaffold count follows the NCBI assembly statistics and excludes organelle assemblies, which are reported separately where present.

Most of the assembly sequence (97.37%) was assigned to 18 chromosomal-level scaffolds. These chromosome-level scaffolds, confirmed by Hi-C data, are named according to size ( Figure 2; Table 3).

**Figure 2.**
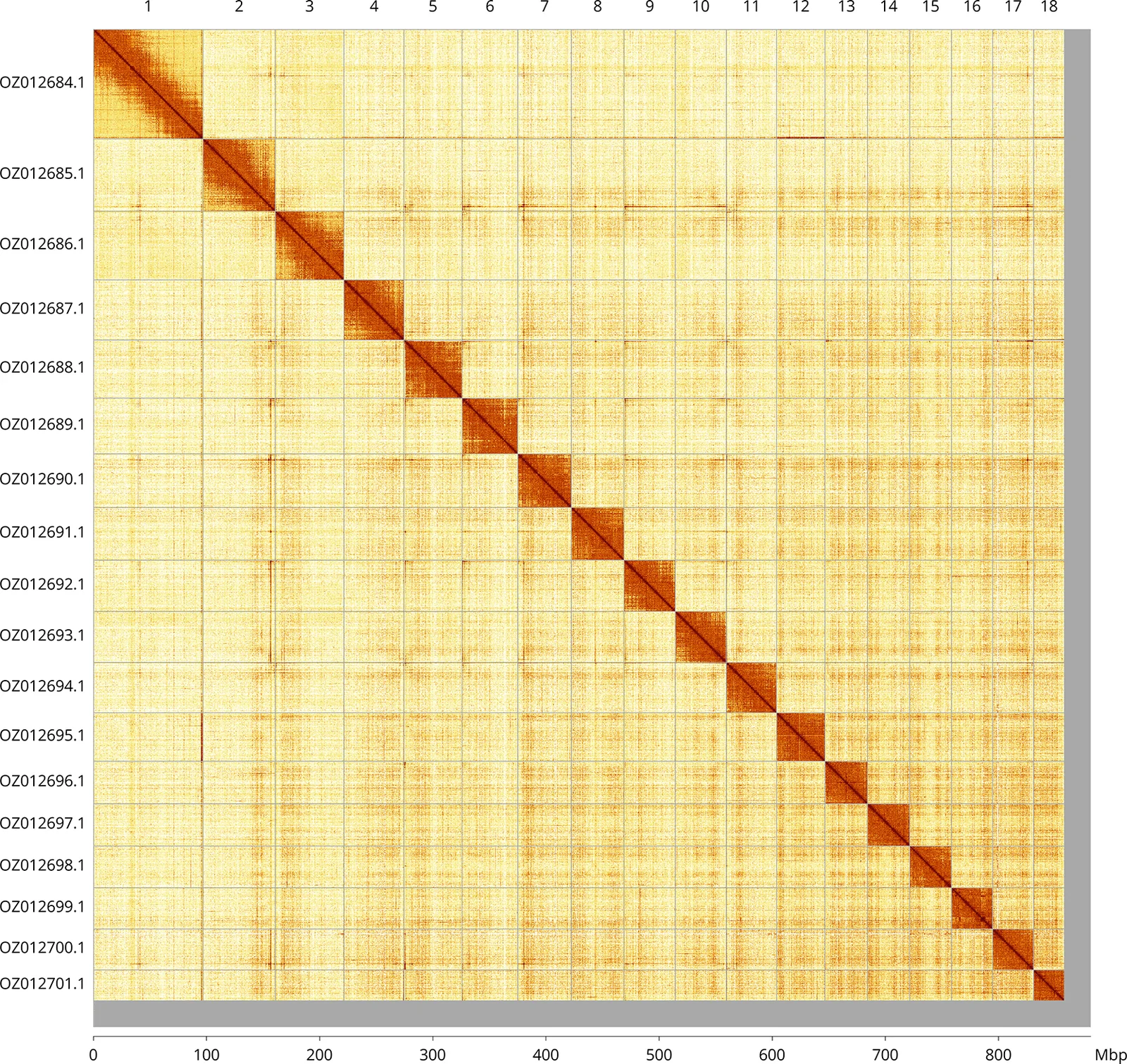
Hi-C contact map of the *Maea johnstoni* genome assembly. Assembled chromosomes are shown in order of size and labelled along the axes, with a megabase scale shown below. The plot was generated using PretextSnapshot.

**Table 3.** Chromosomal pseudomolecules in the primary genome assembly of *Maea johnstoni* wdMagJohn1.

INSDC accession	Molecule	Length (Mb)	GC%
OZ012684.1	1	96.98	37.50
OZ012685.1	2	63.88	37.50
OZ012686.1	3	60.70	37.50
OZ012687.1	4	53.12	37.50
OZ012688.1	5	51.24	37.50
OZ012689.1	6	49.24	37.50
OZ012690.1	7	47.38	37.50
OZ012691.1	8	46.58	37.50
OZ012692.1	9	45.35	37.50
OZ012693.1	10	45.16	37.50
OZ012694.1	11	44.30	37.50
OZ012695.1	12	42.84	37.50
OZ012696.1	13	37.56	37.50
OZ012697.1	14	37.24	37.50
OZ012698.1	15	37.06	37.50
OZ012699.1	16	36.24	37
OZ012700.1	17	36.21	37.50
OZ012701.1	18	27.29	37

The mitochondrial genome was also assembled (length 15.47 kb, OZ012702.1). This sequence is included as a contig in the multifasta file of the genome submission and as a standalone record.

### Assembly quality metrics

The combined primary and alternate assemblies achieve an estimated QV of 49.7. The *k*-mer completeness is 95.62% for the primary assembly, 95.75% for the alternate haplotype, and 99.27% for the combined assemblies.

BUSCO v.5.5.0 analysis using the metazoa_odb10 reference set ( *n* = 954) identified 95.3% of the expected gene set (single = 91.4%, duplicated = 3.9%). The snail plot in Figure 3 summarises the scaffold length distribution and other assembly statistics for the primary assembly. The blob plot in Figure 4 shows the distribution of scaffolds by GC proportion and coverage.

**Figure 3.**
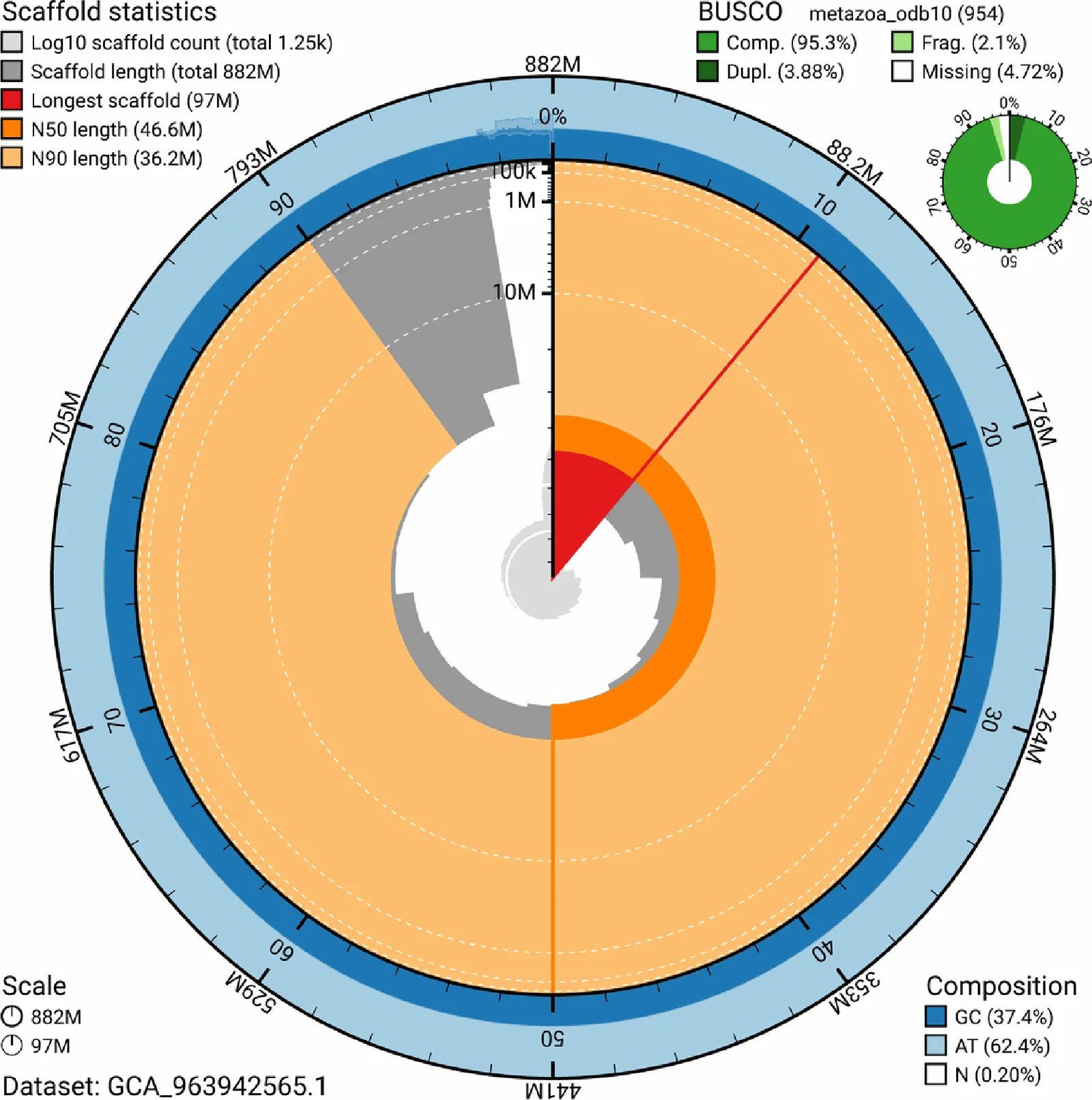
Assembly metrics for wdMagJohn1.1. The BlobToolKit snail plot provides an overview of assembly metrics and BUSCO gene completeness. The circumference represents the length of the whole genome sequence, and the main plot is divided into 1 000 bins around the circumference. The outermost blue tracks display the distribution of GC, AT, and N percentages across the bins. Scaffolds are arranged clockwise from longest to shortest and are depicted in dark grey. The longest scaffold is indicated by the red arc, and the deeper orange and pale orange arcs represent the N50 and N90 lengths. A light grey spiral at the centre shows the cumulative scaffold count on a logarithmic scale. A summary of complete, fragmented, duplicated, and missing BUSCO genes in the metazoa_odb10 set is presented at the top right. An interactive version of this figure can be accessed on the BlobToolKit viewer.

**Figure 4.**
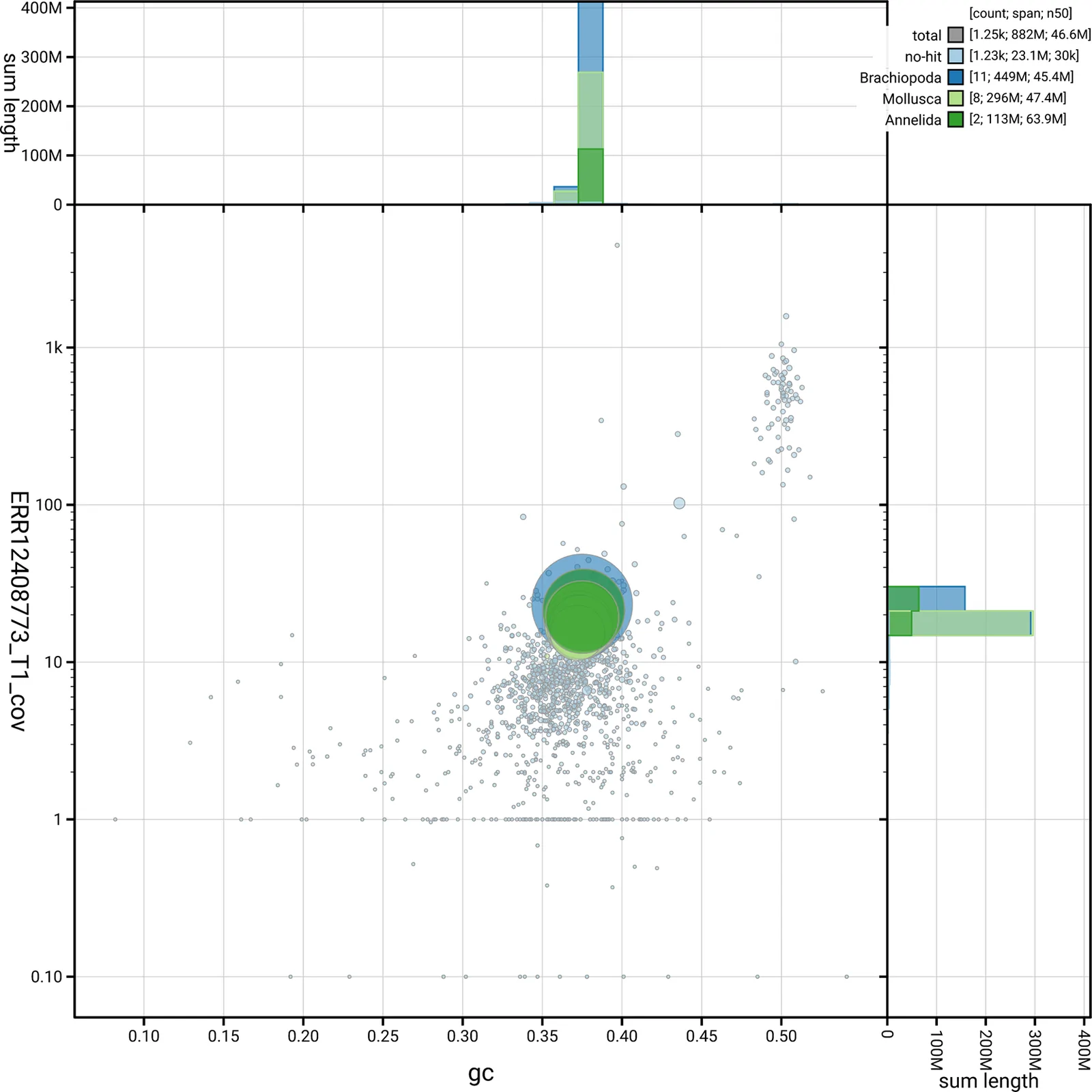
BlobToolKit blob plot for wdMagJohn1.1. The plot shows base coverage (vertical axis) and GC content (horizontal axis). The circles represent scaffolds, with the size proportional to scaffold length and the colour representing phylum membership. The histograms along the axes display the total length of sequences distributed across different levels of coverage and GC content. An interactive version of this figure is available on the BlobToolKit viewer.

Table 4 lists the assembly metric benchmarks adapted from Rhie *et al.* (2021) and the Earth BioGenome Project Report on Assembly Standards January 2026. The EBP metric, calculated for the primary assembly, is **5.C.Q49**.

**Table 4.** Earth Biogenome Project summary metrics for the *Maea johnstoni* assembly.

Measure	Value	Benchmark
EBP summary (primary)	5.C.Q49	6.C.Q40
Contig N50 length	0.14 Mb	≥ 1 Mb
Scaffold N50 length	46.58 Mb	= chromosome N50
Consensus quality (QV)	Primary: 49.3; alternate: 50.2; combined: 49.7	≥ 40
*k*-mer completeness	Primary: 95.62%; alternate: 95.75%; combined: 99.27%	≥ 95%
BUSCO	C:95.3% [S:91.4%, D:3.9%], F:2.1%, M:2.6%, n:954	S > 90%; D < 5%
Percentage of assembly assigned to chromosomes	97.37%	≥ 90%

*Note:* The EBP summary uses log10(Contig N50); chromosome-level (C) or log10(Scaffold N50); Q (Merqury QV). BUSCO: C = complete; S = single-copy; D = duplicated; F = fragmented; M = missing; n = orthologues.

### Metagenome report

We recovered two bins from the metagenome assembly ( Figure 5), one meeting the requirement for a MAG. The recovered bins were both from the phylum Spirochaetota. Table 5 summarises the taxa and quality of the metagenome bins.

**Table 5.** Quality metrics and taxonomic assignments of the binned metagenomes.

NCBI taxon	Taxid	GTDB taxonomy	Quality	Size (bp)	Contigs	Circular	Mean coverage	Completeness (%)	Contamination (%)
uncultured *Spirochaeta* sp.	174390	Spirochaetia	MEDIUM	6782804	33	No	5.56	99.20	7.20
Spirochaetota bacterium	2202144	UBA6919	HIGH	2505751	9	No	5.44	99.34	1.12

**Table 6.** Software versions and sources used for *Maea johnstoni.*

Software	Version	Source
BLAST	2.15.0+	ftp://ftp.ncbi.nlm.nih.gov/blast/executables/blast+/
BlobToolKit	4.3.9	https://github.com/blobtoolkit/blobtoolkit
BUSCO	5.5.0	https://gitlab.com/ezlab/busco
bwa-mem2	2.2.1	https://github.com/bwa-mem2/bwa-mem2
CheckM	2015-01-16	https://ecogenomics.github.io/CheckM/
DAS Tool	1.1.2	https://github.com/cmks/DAS_Tool
DIAMOND	2.1.8	https://github.com/bbuchfink/diamond
dRep	3.4.0	https://github.com/MrOlm/drep
fasta_windows	0.2.4	https://github.com/tolkit/fasta_windows
FastK	1.1	https://github.com/thegenemyers/FASTK
GenomeScope2.0	2.0.1	https://github.com/tbenavi1/genomescope2.0
Gfastats	1.3.6	https://github.com/vgl-hub/gfastats
GTDB-Tk	1.2.1	https://github.com/Ecogenomics/GTDBTk
Hifiasm	0.19.5-r587	https://github.com/chhylp123/hifiasm
HiGlass	1.13.4	https://github.com/higlass/higlass
MerquryFK	1.1.0-c1	https://github.com/thegenemyers/MERQURY.FK
MetaBAT2	2.15-15-gd6ea400	https://bitbucket.org/berkeleylab/metabat
MetaMDBG	Pre-release	https://github.com/GaetanBenoitDev/metaMDBG
Minimap2	2.28-r1209	https://github.com/lh3/minimap2
MitoHiFi	2	https://github.com/marcelauliano/MitoHiFi
MultiQC	1.14; 1.17 and 1.18	https://github.com/MultiQC/MultiQC
Nextflow	23.04.1; 24.10.4	https://github.com/nextflow-io/nextflow
PretextSnapshot	0.0.5	https://github.com/sanger-tol/PretextSnapshot
PretextView	1.0.3	https://github.com/sanger-tol/PretextView
Prokka	1.14.5	https://github.com/tseemann/prokka
purge_dups	1.2.3	https://github.com/dfguan/purge_dups
samtools	1.16.1; 1.17; 1.20; 1.21	https://github.com/samtools/samtools
sanger-tol/ascc	0.1.0	https://github.com/sanger-tol/ascc
sanger-tol/blobtoolkit	0.4.0	https://github.com/sanger-tol/blobtoolkit
sanger-tol/curationpretext	1.4.2	https://github.com/sanger-tol/curationpretext
Seqtk	1.4-r122	https://github.com/lh3/seqtk
Singularity	3.9.0	https://github.com/sylabs/singularity
TreeVal	1.4.0	https://github.com/sanger-tol/treeval
YaHS	1.1a.2	https://github.com/c-zhou/yahs

**Figure 5.**
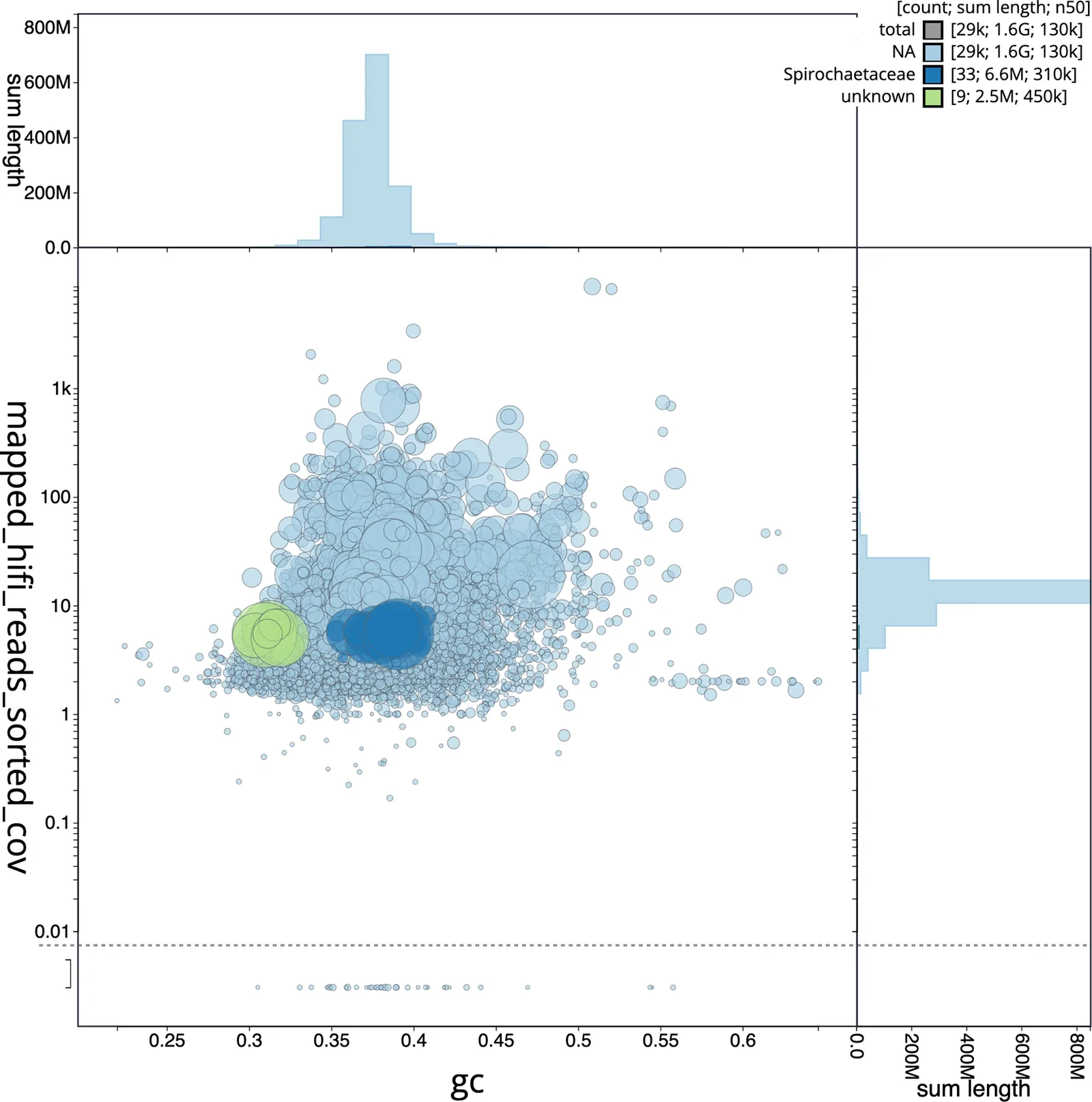
Blob plot for sequences in the *Maea johnstoni* metagenome. The plot shows base coverage (vertical axis) and GC content (horizontal axis). Binned contigs are coloured by family. Circles are sized in proportion to sequence length on a square-root scale, ranging from 510 to 1 274 162. Histograms show the distribution of sequence length sum along each axis. An interactive version of this figure may be viewed here.

## Author information

Contributors are listed at the following links:

• Members of the Wellcome Sanger Institute Tree of Life Management, Samples and Laboratory team
• Members of Wellcome Sanger Institute Scientific Operations – Sequencing Operations
• Members of the Wellcome Sanger Institute Tree of Life Core Informatics team
• Members of the EBI Aquatic Symbiosis Genomics Data Portal Team
• The Aquatic Symbiosis Genomics Project leadership

## Wellcome Sanger Institute – Legal and Governance

The materials that have contributed to this genome note have been supplied by a Tree of Life collaborator. The Wellcome Sanger Institute employs a process whereby due diligence is carried out proportionate to the nature of the materials themselves, and the circumstances under which they have been/are to be collected and provided for use. The purpose of this is to address and mitigate any potential legal and/or ethical implications of receipt and use of the materials as part of the research project, and to ensure that in doing so we align with best practice wherever possible.

The overarching areas of consideration are:

• Ethical review of provenance and sourcing of the material
• Legality of collection, transfer and use (national and international)

Each transfer of samples is undertaken according to a Research Collaboration Agreement or Material Transfer Agreement entered into by the Tree of Life collaborator, Genome Research Limited (operating as the Wellcome Sanger Institute) and in some circumstances other Tree of Life collaborators.

## Data Availability

European Nucleotide Archive: Maea johnstoni. Accession number PRJEB71422; BioProject URL: https://identifiers.org/ena.embl/PRJEB71422. The genome sequence is released openly for reuse. The *Maea johnstoni* genome sequencing initiative is part of the Darwin Tree of Life Project (PRJEB40665), Aquatic Symbiosis Genomics Project (PRJEB43743) and Sanger Institute Tree of Life Programme (PRJEB43745). All raw sequence data and the assembly have been deposited in INSDC databases. The genome will be annotated using available RNA-Seq data and presented through the Ensembl pipeline at the European Bioinformatics Institute. Raw data and assembly accession identifiers are reported in Tables 1 and 2. Production code used in genome assembly at the WSI Tree of Life is available at https://github.com/sanger-tol . Table 6 lists software versions used in this study.
