# Supplementary material for: The chromosomal genome sequence of a shovel-head worm, Maea johnstoni (Fiege, Licher & Mackie, 2000) (Spionida: Magelonidae) and its associated microbial metagenome sequences
Source: Wellcome Open Res. 2026 Jul 21;11:461. [Version 1] doi: 10.12688/wellcomeopenres.27132.1 (PMC13684031; doi:10.12688/wellcomeopenres.27132.1)
Supplement: Peer review history

# Reviewer report: Reviewer response for version 1

**DOI:** 10.21956/wellcomeopenres.29866.r173019

**Published:** 2026-10-20

**Authors:** Joseph Ryan

**Affiliations:** 1 University of Florida, St Augustine, Florida, USA

This paper by Mortimer et al. makes a valuable contribution to invertebrate genomics by presenting the first chromosomal-level genome assembly for Maea johnstoni, a magelonid polychaete worm. the data are of high quality and completeness. The inclusion of associated microbial metagenome sequences further enriches the dataset, making it a useful resource for future comparative and symbiosis research. 

 I had one minor point. The abstract uses the acronym MAG, which is not defined until later in the manuscript. Changing MAG to "metagenome-assembled genomes" would be more clear, especially given that the species is a magelonid.

Are sufficient details of methods and materials provided to allow replication by others?

Yes

Is the rationale for creating the dataset(s) clearly described?

Yes

Are the datasets clearly presented in a useable and accessible format?

Yes

Are the protocols appropriate and is the work technically sound?

Yes

Reviewer Expertise:

evolutionary genomics

I confirm that I have read this submission and believe that I have an appropriate level of expertise to confirm that it is of an acceptable scientific standard.

# Reviewer report: Reviewer response for version 1

**DOI:** 10.21956/wellcomeopenres.29866.r163430

**Published:** 2026-08-26

**Authors:** Erich M Schwarz

**Affiliations:** 1 Cornell University, Ithaca, New York, USA

This is a good genome analysis, and almost entirely satisfactory: I have only one revision to suggest.  The current genus name, for both *M. johnstoni *and *M. mirabilis*, is Maea.  However, all of the cited literature for these species up through 2025, including the 2000 article by Fiege et al. in which *M. johnstoni *was first described, gives them the genus name Magelona.  I found this confusing until I was able to track down a 2026 taxonomy paper not cited in this genome analysis (despite it being written by the genome analysis' lead author!).

 To avoid this confusion, I think it would be very helpful to readers if the following sentence (or one like it) were added to the end of the first paragraph in the Background, and if the 2026 Taxonomy paper by (Mortimer et al.,2026) were added to the References:

 "M. johnstoni and M. mirabilis were both originally assigned to the genus Magelona, but recent morphological and phylogenetic analyses have justified reassigning them to the genus Maea (Mortimer et al., 2026 [Taxonomy 6(1), 17])."

Are sufficient details of methods and materials provided to allow replication by others?

Yes

Is the rationale for creating the dataset(s) clearly described?

Yes

Are the datasets clearly presented in a useable and accessible format?

Yes

Are the protocols appropriate and is the work technically sound?

Yes

Reviewer Expertise:

Nematode genomics

I confirm that I have read this submission and believe that I have an appropriate level of expertise to confirm that it is of an acceptable scientific standard.

## References

[1] : Magelonidae Cunningham & Ramage, 1888 (Annelida, Polychaeta)—Worldwide Identification Keys and the Re-Establishment of Maea Johnston, 1865. *Taxonomy* .2026;6(1) : 10.3390/taxonomy6010017 10.3390/taxonomy6010017
